# bcROCsurface: an R package for correcting verification bias in estimation of the ROC surface and its volume for continuous diagnostic tests

**DOI:** 10.1186/s12859-017-1914-3

**Published:** 2017-11-18

**Authors:** Khanh To Duc

**Affiliations:** Department of Statistical Sciences, University of Padova, via C. Battisti, 241, Padova, 35121 Italy

**Keywords:** Software, R package, ROC surface analysis, Missing at random

## Abstract

**Background:**

Receiver operating characteristic (ROC) surface analysis is usually employed to assess the accuracy of a medical diagnostic test when there are three ordered disease status (e.g. non-diseased, intermediate, diseased). In practice, verification bias can occur due to missingness of the true disease status and can lead to a distorted conclusion on diagnostic accuracy. In such situations, bias–corrected inference tools are required.

**Results:**

This paper introduce an R package, named *bcROCsurface*, which provides utility functions for verification bias–corrected ROC surface analysis. The shiny web application of the correction for verification bias in estimation of the ROC surface analysis is also developed.

**Conclusion:**

*bcROCsurface* may become an important tool for the statistical evaluation of three–class diagnostic markers in presence of verification bias. The R package, readme and example data are available on CRAN. The web interface enables users less familiar with R to evaluate the accuracy of diagnostic tests, and can be found at http://khanhtoduc.shinyapps.io/bcROCsurface_shiny/.

## Background

The use of diagnostic tests is becoming more and more popular in medicine, a popularity that feeds the need for assessing their accuracy. A common approach employed to this aim is receiver operating characteristic (ROC) analysis. For a three class disease status (e.g., non-diseased, intermediate and diseased), the ROC surface and the volume under ROC surface (VUS) are frequently used. The graph of ROC surface lies in the unit cube and the VUS varies from 0 to 1. More precisely, the ROC surface of a useless test is the plane of the triangle with three vertices (1,0,0), (0,1,0) and (0,0,1), whereas the ROC surface corresponding to a perfect test is the surface of the unit cube. Consequently, the value of VUS is 1/6 in case of useless tests, and 1 if the diagnostic test is perfect.

There are various methods [1] for estimating a ROC surface and its VUS when all subjects in the study undergo a gold standard (GS) test, a condition often referred to as full verification of subjects. In R, some packages exist for ROC surface analysis under full verification. For example, *DiagTest3Grp* [2] gives some tools for estimating VUS, *ROCS* [3] deals with the high-throughput class-skewed data and *HUM* [4] provides tools for visualizing the ROC surface.

Usually, however, only a subset of subjects is selected to undergo disease verification, due to the expensiveness and/or invasiveness of the GS test. If only the verified subjects are used for estimating the ROC surface and VUS, inference tools are biased, an effect known as verification bias. No package is available for correcting for verification bias estimators of the ROC surface and VUS. The R package *bcROCsurface* aims at filling this gap. It provides several new functions for bias-corrected ROC surface analysis. More precisely, it implements methods in To Duc et al. [5, 6], who proposed five bias-corrected estimators for ROC surface and VUS of a continuous diagnostic test, namely, full imputation (FI), mean score imputation (MSI), inverse probability weighting (IPW), semiparametric efficient (SPE) and K nearest-neighbor (KNN) estimators. These methods perform provided that the missing mechanism is MAR (missing at random).

## Implementation

The *bcROCsurface* imports various R packages (e.g., rgl, nnet, boot) and is built on Rcpp [7]. The package is freely available to download from CRAN - a global repository of R packages http://cran.r-project.org, and provides several functions for bias–corrected inference on VUS, for constructing and plotting 3D-ROC surfaces as well as ellipsoidal confidence regions of true class fractions at a given cut-point.

The data to be elaborated should include: a variable representing the disease status (categorical variable with three classes), a variable representing the diagnostic test (continuous variable) and a variable representing the verification status (binary variable, 1 and 0 indicate verified and not verified subject, respectively). Some other auxiliary covariates (numeric variables) may also be present. Practical use of the package foresees three steps: data preparation, modeling and inference.

### Data preparation

In this step, the condition of monotone ordering of the three disease classes under study [8] is checked. The condition is mandatory to perform the subsequent analyses. In words, the condition assumes that subjects from class 3 have higher test results than subjects in class 2 and the latter have higher test results than subjects in class 1. Function preDATA() performs such checks, warning users in case monotone ordering is not satisfied. When satisfied, the function coerces the disease status in the numeric format (1, 2, 3) corresponding to increasing disease status. It also generates a binary matrix with three columns, corresponding to the coding of the three classes of the disease status, used as input of the main functions.

### Modeling

Correction for verification bias requires estimation of a disease and a verification model. The function psglm() obtains the verification probabilities specifying a general linear model for the verification process. In practice, the user can select among a logistic, a probit or a threshold regression model (default is logistic model). Functions rhoMLogit() and rhoKNN() estimate the disease probabilities based on a multinomial logistic regression. In particular, rhoMLogit() calls the *nnet* package for multinomial logistic modeling, whereas rhoKNN() uses K nearest-neighbor regression.

### Inference

Two main functions are provided: ROCs() for constructing and plotting ROC surfaces, and vus() for estimating VUS values as well as obtaining confidence intervals. Estimation methods can be flexibly selected by the argument method, among 6 options, i.e., full if the full data is available; fi for the FI estimator, msi for the MSI estimator, ipw for the IPW estimator, spe for the SPE estimator and knn for the KNN estimator in presence of partial verification (see [5, 6] for the definition of the estimators). To plot ROC surfaces and ellipsoid confidence regions, the function ROCs() employs the plotting functions of the *rgl* package. vus() employs some core functions, written in the C++ language and integrated in R through the *Rcpp* and *RcppArmadillo* packages. Confidence intervals of VUS values are built based on the asymptotic distribution or the bootstrap resampling process (supported by the parallel computing). In addition, this function also performs the statistical test, H_0_: VUS = 1/6 versus H_1_: VUS > 1/6. The test statistic is

$$t = \frac{\widehat{\text{VUS}} - 1/6}{\sqrt{{\widehat{\text{Var}}(\widehat{\text{VUS}})}}} \overset{.}{\sim} \mathcal{N}(0,1), $$

where $\widehat {\text {VUS}}$ is the estimator of VUS and $\widehat {\text {Var}}(\widehat {\text {VUS}})$ denotes the estimate of the variance of $\widehat {\text {VUS}}$.

Besides the functions described above, the package also offers other functions for estimating variances and for choosing *K* to compute the KNN estimate.

**Fig. 1:**
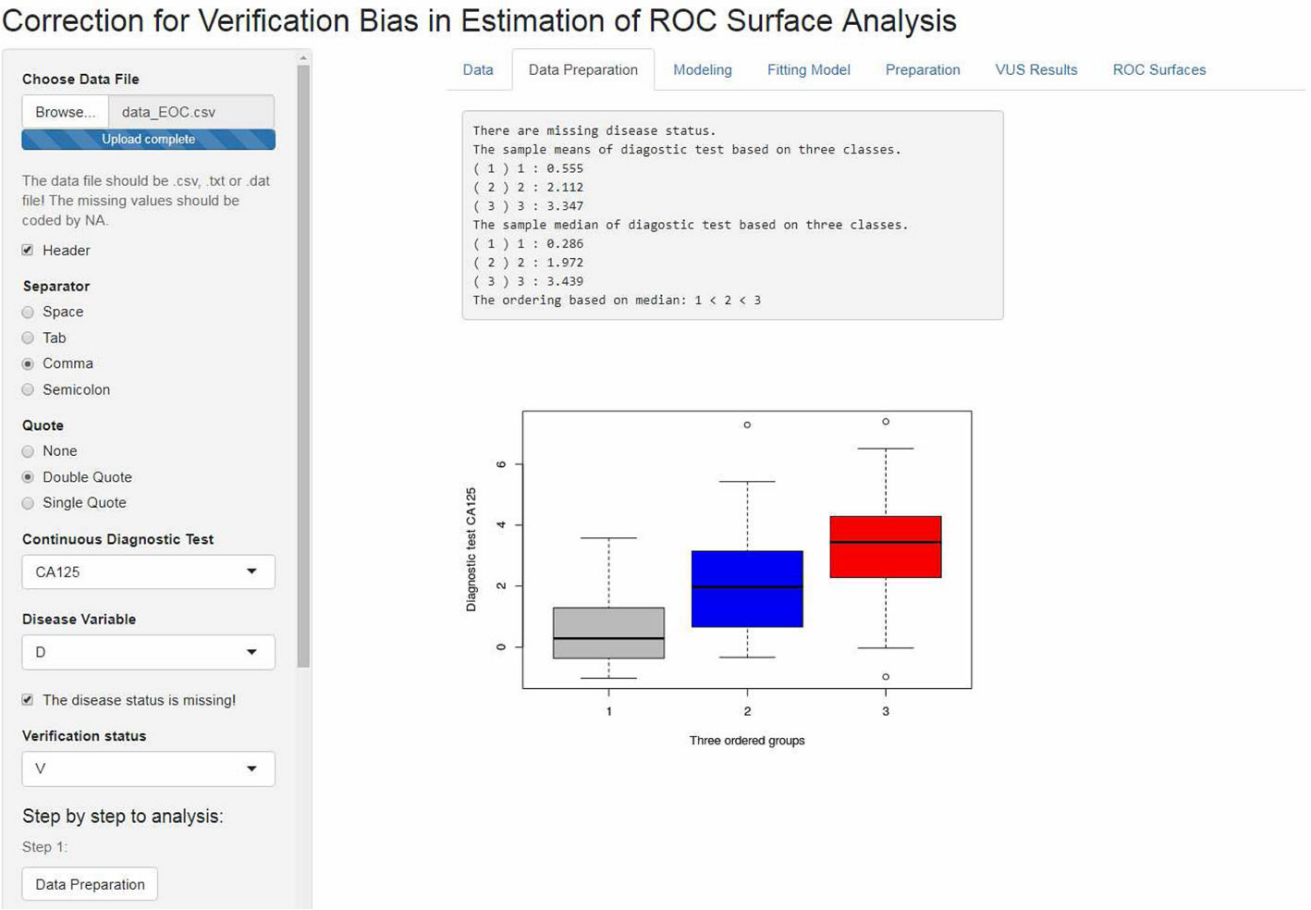
Screenshot of the GUI for bcROCsurface, built in shiny web application. The boxplot of diagnostic test results corresponding to three ordered disease status

A Shiny web application has also been developed to provide the possibility to deploy *bcROCsurface* package over the web. The layout of the bcROCsurface web interface is clean and straightforward (Fig. 1). It provides the possibility to load the datasets for the analysis and to access all functions of *bcROCsurface* package. Here, the user loads a data file (typically,.csv,.txt or.dat file), selects a suitable option for “Separator” and “Quote” to read data correctly, then chooses the input variables, i.e. diagnostic test, disease status. If the true disease status is not missing, the user follows step 1 and 2 to get the results. Otherwise, the user clicks on the square box and selects the verification status, then follows step 1, 2 and 3 to implement the bias-corrected ROC surface analysis.

## Results and discussion

In the following example, the package is employed to evaluate the accuracy of ovarian cancer-related tumor marker CA125 (cancer antigen 125), which is a highly glycosylated sialomucin that is expressed on epithelial cell surface, especially on ovarian cancer cells. The dataset, available in the package, is described in detail in [5]. In what follows, CA125 will be the diagnostic test of interest and CA153 and Age will be two auxiliary covariates. Three classes of cancer are considered, i.e., benign (1), early stage (2) and late stage (3). The first six lines of the dataset are shown below (V denotes verification status and D disease status).



As mentioned above, in the first step, the application of preDATA() is needed to ensure that the package can be employed. In the second step, to produce estimators FI, MSI, IPW and SPE, the functions rhoMLogit() and psglm() are called to fit the disease model and verification model. Finally, vus() is used to obtain the bias-corrected estimates of VUS for marker CA125 and the values of the statistic *t* for testing H_0_: VUS = 1/6 vs. H_1_: VUS > 1/6. Results were produced using R code below.





Table 1 shows the four bias-corrected estimates of VUS, associated standard error and approximate 95% confidence intervals built with and without logit transformation. Table 2 shows the values of *t*-stat as well as *p*-values for testing H_0_: VUS = 1/6 vs. H_1_: VUS > 1/6. Statistical interpretation of results in Table 1 is given in [5], Section 5.1. Being this an artificial example, i.e., an example in which missingness of the disease has been artificially created according to the MAR assumption, the above mentioned section explains how to evaluate usefulness of bias correction and why SPE and IPW can be considered good estimates in this case.

**Table 1:** The bias-corrected estimates of VUS and corresponding 95% confidence intervals built with and without logit transformation

	Estimate	Std. Err	Lower. Normal	Upper. Normal	Lower. Logit	Upper. Logit
FI	0.5150	0.0404	0.4357	0.5942	0.4360	0.5932
MSI	0.5183	0.0415	0.4368	0.5997	0.4371	0.5985
IPW	0.5500	0.0416	0.4685	0.6314	0.4679	0.6294
SPE	0.5581	0.0443	0.4712	0.6450	0.4703	0.6424

**Table 2:** Testing hypothesis, H_0_: VUS = 1/6 vs H_1_: VUS > 1/6

	t-stat	*p*-value
FI	8.6168	< 0.0001
MSI	8.4644	< 0.0001
IPW	9.2212	< 0.0001
SPE	8.8270	< 0.0001

**Fig. 2:**
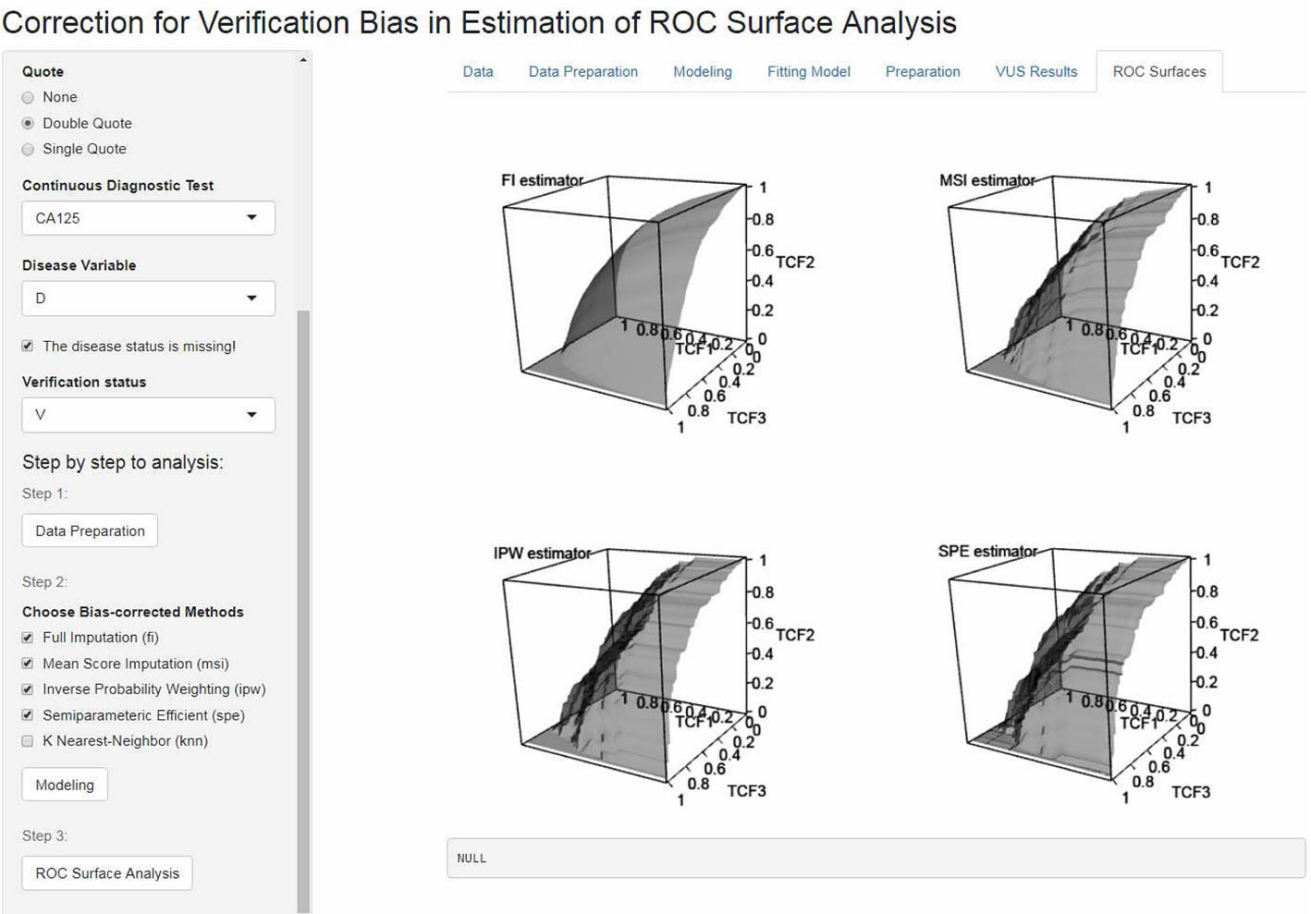
Bias-corrected ROC surfaces in Shiny application. Full imputation (FI), Mean score imputation (MSI), Inverse probability weighting (IPW) and Semiparametric efficient (SPE) estimators are implemented to estimate ROC surface

The shiny web application is also easy to use. By using it, the four bias-corrected ROC surfaces of biomarker CA125, described above, can be easily obtained and are shown in Fig. 2.

From a computational point of view, the demand of *bcROCsurface* is essentially associated to estimation of variances of VUS (see [5]). To establish the computation time of the functions asyVarVUS() and vus(), a simulation study is performed. The disease status is generated as a trinomial random vector (*D*_1_,*D*_2_,*D*_3_), such that *D*_*k*_ is a Bernoulli random variable with mean *θ*_*k*_, *k*=1,2,3. Setting *θ*_1_=0.4,*θ*_2_=0.35 and *θ*_3_=0.25. A diagnostic test *T* and an auxiliary covariate *A* are generated from the following conditional models

$$T,A |D_{k} \sim \mathcal{N}_{2} \left(k \left({2 \atop 1}\right), \left(\begin{array}{cc} 1.75 & 0.1 \\ 0.1 & 2.5 \end{array}\right) \right), \ \ \quad k = 1,2,3. $$

The verification status *V* is simulated by using the following model

$$\text{logit}\left\{\Pr(V = 1|T,A)\right\} = 1 - 2.2 T + 4 A. $$

**Fig. 3:**
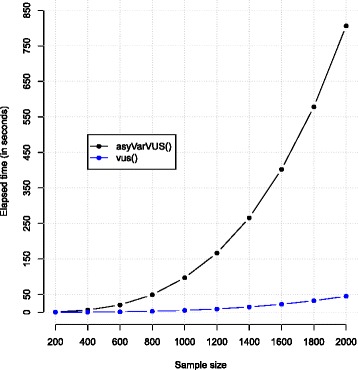
Computation time of asyVarVUS() and vus() for the SPE estimator

In this simulation, the SPE estimator is employed (being the most computationally demanding) and the sample size varies from 200 to 2000. The computation is replicated 100 times and is performed on a PC Intel(R) Core(TM) i7-2720QM CPU, 2.2 GHz, 8.00 GB RAM. The average elapsed times (in seconds) of the functions vus() and of asyVarVUS() are shown in Fig. 3.

## Conclusions

The paper presents the R package *bcROCsurface*, that provides routines to construct the ROC surface and estimate the VUS for continuous diagnostic tests when disease status is missing at random. The shiny web interface is straightforward to use and is therefore accessible to users less familiar with the programming language R.

## Availability and requirements

The bcROCsurface package is available on CRAN (http://CRAN.R-project.org/package=bcROCsurface), which is compatible with any operating system supporting R program. The license is GPL-2 | GPL-3. The Shiny web application is freely available to all users from http://khanhtoduc.shinyapps.io/bcROCsurface_shiny/.

## Abbreviations

FI: Full imputation
IPW: Inverse probability weighting
KNN: K nearest-neighbor
MAR: Missing at random
MSI: Mean score imputation
ROC: Receiver operating characteristic
SPE: Semi-parametric efficient
VUS: Volume under ROC surface
